# Study on the Selectivity of Molecular Imprinting Materials Determined through Hydrogen Bonding on Template Molecular Structures of Flavonoids

**DOI:** 10.3390/molecules29061292

**Published:** 2024-03-14

**Authors:** Siyue Guan, Yue Wang, Ting Hu, Lingling Che, Xiaoqiao Wang, Yike Huang, Zhining Xia

**Affiliations:** 1 School of Pharmaceutical Sciences, Chongqing University, Chongqing 401331, China; 2 College of Pharmacy, Chongqing Medical University, Chongqing 400016, China

**Keywords:** flavonoid, hydrogen bond, molecular imprinting, selectivity, intensity of interaction

## Abstract

Molecular imprinting technology is widely used for the specific identification of compounds, but the selective recognition mechanisms of the same compounds still need to be further studied. Based on differences in hydrogen bond size and orientation, molecularly imprinted polymers (MIPs) were designed to adsorb flavonols with the same parent core and different hydroxyl groups. A surface-imprinted material was designed with silicon dioxide as the carrier, myricetin as the template molecule, and methacrylic acid (MAA) as the functional monomer. Scanning electron microscopy (SEM), Brunauer–Emmett–Teller surface area (BET) analyses, Fourier-transform infrared spectroscopy (FT-IR), and other characterization experiments were carried out. The intrinsic mechanism of the MIPs was also explored. The MIPs showed good adsorption of myricetin and other flavonoids through hydrogen bonding and steric hindrance. The adsorption capacity was 3.12–9.04 mg/g, and the imprinting factor was 1.78–3.37. Flavonoids with different hydroxyl groups in different numbers and directions had different hydrogen bond strengths with functional monomers. R_2_, R_4_, and R_1_ on 2-phenylchromogenone had stronger electronegativity, and the hydroxyl group was also more likely to form and have stronger hydrogen bonds. The hydroxyl negativity and the degree of steric hindrance of flavonoids played a major role in the recognition of molecularly imprinted materials. This study is of great significance for the synthesis of and selection of templates for analogous molecular imprinting materials.

## 1. Introduction

Molecular imprinting technology is based on the interaction between template molecules and functional monomers to prepolymerize and then polymerize through cross-linking agents to form polymers [1,2]. A template is eluted to form recognition materials that can reflect the shape and interaction of the template molecules. The resultant molecularly imprinted polymers have holes with multiple action points that match the spatial configuration of the template molecules, and such imprinted cavities have specific selectivity for the template molecules [3]. In recent years, MIPs have been studied in the fields of solid-phase extraction (SPE) [4], chromatographic separation [5], chemical sensors [6], and artificial antibodies [7] due to their low cost, easy preparation, good adsorption dynamics, and good imprinting effects. Applications in environmental monitoring, bioanalysis, and food testing—especially when using MIPs as adsorbents for sample pretreatment—have attracted much attention in terms of preconcentration and selective enrichment [3,8].

Flavonoids are a class of natural compounds with the structure of 2-phenylchromogenone. The parent core of a flavonoid often contains hydroxyl, methoxy, hydrocarbon, isopentenoxy groups, as well as other substituents [9]. Because they are widely present in various plants, they exert a variety of effects, such as anti-cancer [10], anti-inflammatory [11], and antibacterial properties [12,13], as well as cardiovascular protection [9,14,15]. Therefore, flavonoids have a high health promotion value. to the means of extracting and isolating flavonoids from plants have attracted considerable interest from scientists, and researchers have conducted many studies on molecular imprinting materials for this class of compounds [16,17]. Xia et al. [18] prepared novel MIP microspheres through precipitation polymerization using 4-vinylpyridine (4-VP) as a functional monomer and ethylene dimethacrylate (EGDMA) as a crosslinker for the selective extraction of kaempferol. Yang et al. [19] prepared a virtual molecularly imprinted polymer using naringenin as a virtual template for the selective enrichment of flavonoids. Liu et al. [20] designed a one-step RAFT Pickering HIPE emulsion blotting method to prepare a porous hydrophilic boric acid affinity blotting hydrogel for the specific separation and purification of naringin. However, researchers generally focused on the selective recognition of a specific compound by molecular imprinting materials and conducted experiments to screen template molecules or select functional monomers, and they rarely discussed or studied the adsorption performance and interaction mechanism of a class of compounds at a deeper level [21]. Therefore, more theoretical studies need to be conducted to develop research on MIPs.

In this work, we present a strategy for investigating the ability to selectively recognize flavonoids using hydrogen bonding. An imprinted material that is synthesized using myricetin as a template can be recognized through hydrogen bonding between the hydroxyl group on 2-phenylchromogenone and the functional monomer. In addition, myricetin has more hydroxyl groups on the 2-phenylchromogenone structure, which helps demonstrate its hydrogen bond recognition mechanism more clearly at the molecular level. 

In this work, imprinted materials were prepared via surface-imprinted polymerization using myricetin as the template molecule, methacrylic acid (MAA) as the functional monomer, acetonitrile as the pore-forming agent and solvent, and EGDMA as the crosslinking agent. A series of adsorption experiments were performed to investigate the material and chemisorption properties of the MIPs. In addition, the ability of the MIPs to adsorb to flavonol compounds with different numbers or sites of hydroxyl groups containing 2-phenyl chromogenic ketone as the core structure was studied. The differences in the abilities of imprinted materials to adsorb to a series of flavonoids were compared and analyzed in order to determine the effects of hydrogen bond strength and position on adsorption selectivity. This study could provide a reference for the improvement of MIPs’ selectivity for compounds, as well as the reasonable selection of templates and synthesis schemes for quasi-selective MIP studies.

## 2. Results and Discussion

### 2.1. Preparation and Characterization of MIPs

There are many methods for the synthesis of the MIPs of flavonoids. The main methods are bulk polymerization, surface imprinting, and precipitation polymerization. In order to balance the simplicity of synthetic materials and improve their selectivity, the surface imprinting method was selected in this study, and the commonly used SiO_2_ was used as the carrier. MAA was used as a functional monomer, and myricetin was deliberately chosen as a template molecule. MAA can hydrogen bond with the hydroxyl group of myricetin. In order to investigate the effects of the number and sites of hydroxyl groups, the present study focused on the effect of the ratio of functional monomers and crosslinkers on MIP synthesis. Due to the free radical polymerization reaction of the functional monomer and crosslinker for synthesizing MIPs under the action of azobisisobutyronitrile (AIBN), under the same conditions, different proportions of crosslinker affect the looseness of the material, different proportions of functional monomer affect the interaction between the hydroxyl group of template molecule and the functional monomer, and both of them affect the number of template molecule binding sites on MIPs. By varying the myricetin–MAA–EGDMA ratio, nine materials were synthesized, as shown in Table 1. From the table, one can clearly see the adsorption quantity (Q) and imprinting factor (IF) of the MIPs. The prepared MIPs (MIP 2) showed good adsorption (Q = 15.13 mg/g) and good imprinting (IF = 1.85) at a molar ratio of myricetin–MAA–EGDMA of 1:5:30, and the material synthesized at this ratio was used for the subsequent experiments.

In addition, in the selection of a porogenic agent, the MIPs synthesized using acetonitrile (ACN), methanol (MeOH), and tetrahydrofuran (THF) were chosen for comparison. It can be seen that there were obvious differences in the IF; the MIPs synthesized using ACN as a porogen had the best effect. Since the properties of molecule-imprinted polymers, such as swelling and porosity, are often related to the solvent conditions used in the polymerization process, the properties of the porogen affect the selectivity and efficiency of MIPs. The pore-causing agent selected should be conducive to both the formation of interactions and the formation of porous structures. Although THF is a non-polar solvent and contributes to the formation of interactions, due to its structure and physical properties, the materials synthesized using THF are very dense, which is not conducive to the adsorption of materials. Compared with ACN, the IF value of MeOH as a porogen is very small. It is the greater polarity of MeOH than that of ACN that affects the strength of its interaction with imprinted molecules.

**Figure 1:**
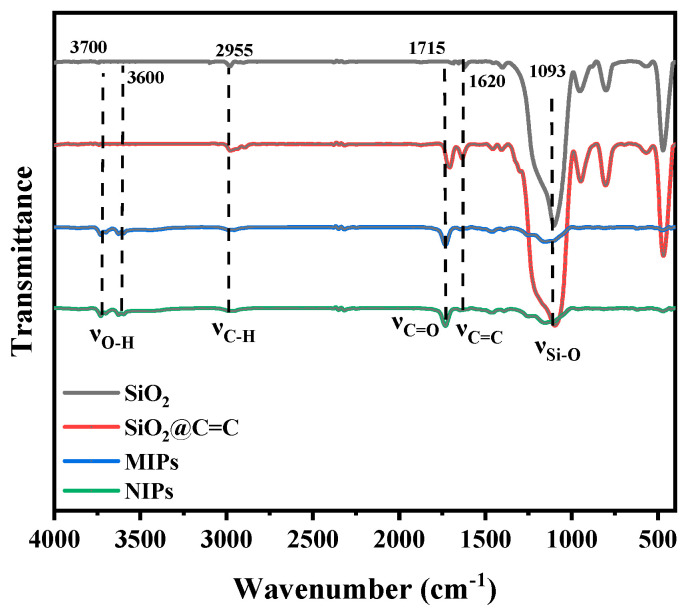
Fourier-transform infrared spectra of SiO_2_, SiO_2_@C=C, MIPs, and NIPs.

The infrared spectral data of SiO_2_, SiO_2_@C=C, MIPs, and NIPs were collected, as shown in Figure 1. A strong peak at 1093 cm^−1^, ν_Si–O_, was observed in several material samples, while the peaks of the MIPs and NIPs were weakened at this point, indicating the successful polymerization of the crosslinker on the support. Moreover, compared with SiO_2_, the other samples had a peak at 1620 cm^−1^, ν_C=C_, which indicated that the double bond was successfully modified on SiO_2_, and the peak at 1715 cm^−1^ was that of ν_C=O_, which also indicated that the grafting of the silane coupling agent (KH-570) was successful. In addition, the two small-envelope peaks at 3600 and 3700 cm^−1^ for MIPs and NIPs were ν_O–H_, unlike SiO_2_ and SiO_2_@C=C, which may have accounted for the introduction of functional monomers or crosslinkers in the polymerization process. Finally, since the chemical composition of the MIPs and NIPs was the same and the template molecules were completely eluted, there were no significant differences in the Fourier-transform infrared spectra. 

**Figure 2:**
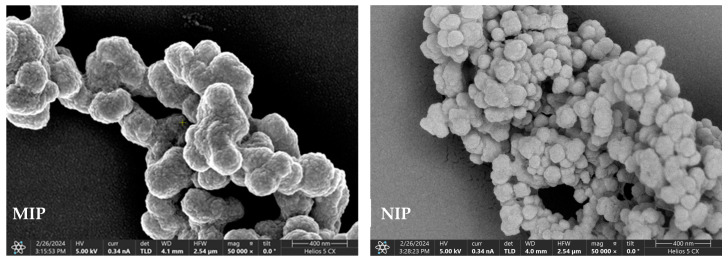
Scanning electron microscope images of MIPs and NIPs.

Figure 2 shows images of the MIPs and NIPs obtained through scanning electron microscopy (SEM). In the figure, one can clearly see that the MIPs and NIPs had obvious differences in morphology, size, and other aspects. The MIPs ranged from 150 to 200 nm, and the NIPs ranged from 90 to 140 nm. The surface of the MIPs was rougher than that of the NIPs, which may have been because the MIPs synthesized by adding template molecules left many imprinted cavities after elution from the template. In contrast, NIP synthesis was performed without the addition of template molecules, and there were no specific cavity structures and significantly smaller particles.

**Figure 3:**
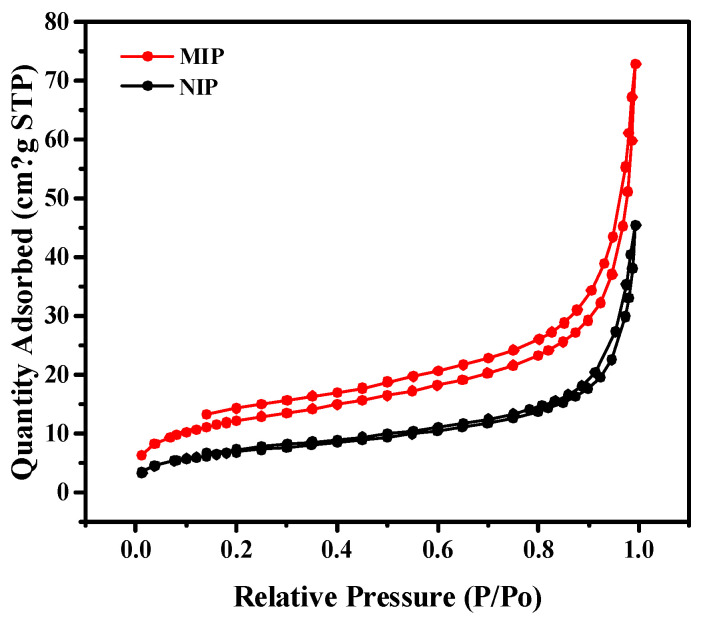
Nitrogen adsorption and desorption isotherms of MIPs and NIPs.

Figure 3 shows that the adsorption isotherms of both the MIPs and NIPs were H2-type hysteresis loops with mesoporous structures. The specific surface area of the MIPs was 60.90 m^2^/g. Compared with the NIPs at 34.88 m^2^/g, the MIPs had a larger specific surface area and more imprinting sites. The pore volumes of the MIPs and NIPs were 0.11 cm^3^/g and 0.07cm^3^/g, respectively, which further indicated that the MIPs had more specific adsorption holes. In addition, the pore width of the MIPs (10.99 nm) was smaller than that of the NIPs (14.29 nm).

### 2.2. Adsorption Properties

#### 2.2.1. Effect of Solvent Polarity on the Synergistic Action of MAA in MIPs

**Figure 4:**
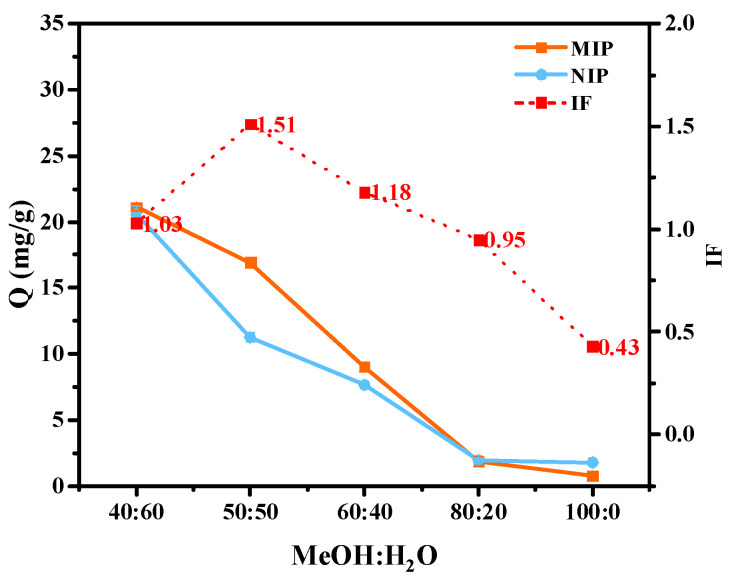
Effect of the solvent on the adsorption and IF of myricetin by MIPs and NIPs.

The adsorption solvent had a significant influence on the adsorption effect, and the following were the results of the adsorption experiment with different proportions of MeOH and H_2_O as the adsorption solvent. Figure 4 shows that the adsorption also exhibited an overall decrease with the decrease in the H_2_O ratio, but when the MeOH:H_2_O ratio was in the range of 40:60 to 50:50 (*v*/*v*), the IF of the rapidly MIPs increased and then decreased from 50:50 to 100:0 (*v*/*v*). As for the finding that the adsorption capacity gradually decreased with the increase in MeOH content, this was because the imprinted molecules were soluble in MeOH but not easily soluble in water. When the water content was higher, the imprinted molecules tended to enter the MIPs, and the adsorption capacity was higher. In addition, solvent experiments with higher water ratios were not performed, although a higher water content resulted in greater adsorption because the water compatibility of the flavonoids was very poor, and they were almost insoluble in pure water. 

It can also be seen in Figure 4 that the IF value showed an inflection point at an equal ratio of MeOH:H_2_O (*v*/*v*, 50:50). Firstly, in the solvent environment with MeOH/H_2_O, a high water content was conducive to the enhancement of hydrophobic interactions, which were beneficial to the entry of imprinted molecules. However, as a highly polar solvent, water also affected the weak interactions of hydrogen bonds. Under these conditions with a high proportion of water, the hydrophobic interactions were strong, whereas the hydrogen bonding was weak, and the less-water-soluble flavonoids also tended to bind to the MIPs in a non-specific manner. Thus, these flavonoids had higher adsorption capacity in MeOH and H_2_O (*v*/*v*, 40:60) but not the highest IF value. With the decrease in the water content in the adsorption solvent, the interference of the water molecules in the hydrogen bond interactions between MeOH and MAA was less significant, the hydrogen bond interactions were enhanced, and the specific binding ability of the MIPs was enhanced. At this time, although the amount of adsorption decreased, the IF value increased. However, as the amount of methanol increased, the hydrophobic interactions continued to decrease. At this point, the imprinted molecules were more soluble in MeOH rather than binding to the MIPs through hydrogen bonding. Therefore, both Q and IF showed a downward trend. Even higher than a ratio of MeOH:H_2_O of 80:20 (*v*/*v*), IF < 1 may also have been due to the enhancement of non-specific binding, and there were more binding sites of non-imprinted holes on the surface of NIPs than on that of MIPs. 

#### 2.2.2. Effect of pH on Hydrogen Bonds 

**Figure 5:**
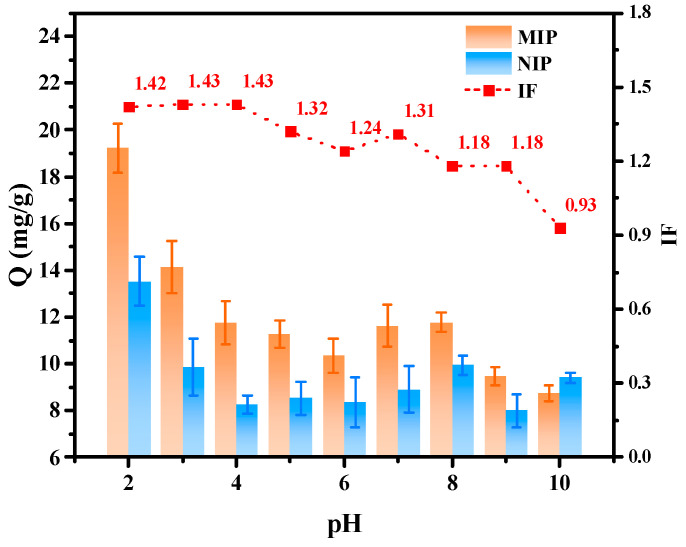
Adsorption and IF of myricetin on MIPs and NIPs and their changes with pH.

Figure 5 shows the Q and IF of myricetin with the MIPs and NIPs in the adsorption solvent (MeOH:H_2_O = 50:50) at different pH levels. As the pH increased, the IF decreased as a whole, but it increased at pH = 7 and then began to decrease again. Firstly, the myricetin adsorption ability of the MIPs was the highest at pH = 2, as with other flavonoids (S1), and the adsorption ability of the MIPs increased with the increase in acidity. This is because, in acidic solutions, flavonoids mainly exist in molecular form, and their polyhydroxy structure is more likely to combine with MAA to form hydrogen bonds. However, with the increase in the pH from 4, the carboxyl group of MAA (pKa = 4.66) may have been partially or completely ionized and negatively charged. At this time, myricetin (pKa = 6.30) was also partially ionized. This negatively charged state affected the hydrogen bond interactions between MAA and the imprinted molecules, thereby affecting the adsorption effect. In this experiment, this resulted in a gradual decrease in the adsorption capacity of the material with increasing pH (pH 4–6), as well as a decrease in IF. However, when the pH was between 6 and 7, the IF of all flavonoids showed an inflating point because when the pH gradually increased, the OH^−^ content of the flavonoids increased, the hydrogen bond with MAA strengthened, and the increase in specific binding led to an increase in the IF value. However, with the further increase in alkalinity (pH > 7), although the hydroxyl group of flavonoids was also ionized in the negative state, the amount of OH^−^ in the solvent also increased, and the carboxylic acid of MAA was more likely to form hydrogen bonds with the solvent, which weakened the hydrogen bond between the template molecule and the functional monomer, and the IF value gradually decreased with the increase in pH. The adsorption results of this material for other flavonoids were similar. In order to determine the appropriate pH of the material for other flavonoids at the same time although the MIPs had a better adsorption effect on myricetin at pH = 4 or 7 and to realize the differences in the abilities of MIPs to adsorb to a series of flavonoids, the condition of pH = 6 was selected for the subsequent experiments.

#### 2.2.3. Binding Characteristics of MIPs and NIPs

**Figure 6:**
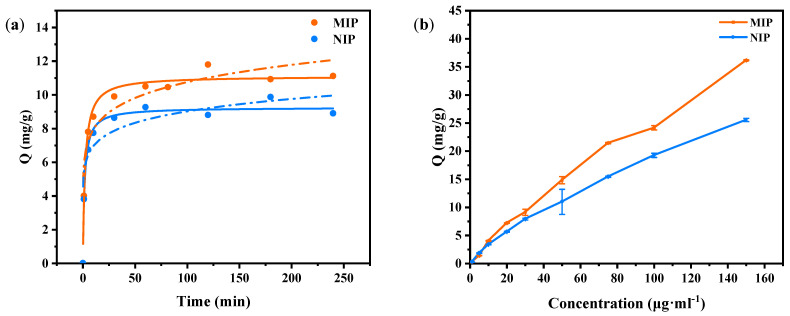
(**a**) Kinetic curves for adsorption of myricetin by the MIPs and NIPs, the solid line is the Langmuir fit curve and the dashed line is the Freundlich fit curve; (**b**) isothermal adsorption curves of MIPs and NIPs for myricetin.

Figure 6a shows the kinetic curves of the adsorption of myricetin by the MIPs and NIPs. Analysis of the Langmuir and Freundlich isothermal models was performed using the static adsorption of the MIPs and NIPs. The adsorption type of the MIPs and NIPs was more consistent with the Langmuir adsorption isotherm formula. It can be seen in the figure that the two curves had the same trend, but they had obvious differences in the adsorption amount, which was mainly due to their structure. There were large numbers of imprinted holes on the surface of the MIPs synthesized by adding template molecules through the surface imprinting method, and they had a high affinity with the template molecules. These holes were able to quickly adsorb imprinted molecules (0–10 min) and could reach a high adsorption capacity in a short time. As the adsorption time passed, the imprinted holes were gradually occupied, and the adsorption rate gradually decreased. Finally, adsorption equilibrium was reached at 120 min, and the adsorption capacity reached 11.80 mg/g. In contrast, the synthesis process for NIPs did not include the addition of template molecules, so there were no specific cavity structures on the surface to combine with myricetin molecules. The functional monomers were arranged disorderedly in the imprinted layer on the surface. Although the imprinted molecules also bound to it, the adsorption of the NIPs to myricetin was non-specific, with a low adsorption rate and low adsorption capacity. Moreover, because there were no specific hole structures on the surface of the NIPs, the mass transfer resistance of the template molecules was small; the time taken to reach adsorption equilibrium was relatively short at 60 min, and the adsorption amount was 9.27 mg/g.

Figure 6b shows the myricetin adsorption isotherms of the MIPs and NIPs. Firstly, it can be clearly seen that the adsorption isotherms of the MIPs exhibited a higher adsorption capacity and a steeper curve shape than those of the NIPs. This was because the MIPs formed imprinted holes on their surfaces that were highly matched with the shape, size, and functional groups of the template molecules through the imprinting process, and these holes could specifically adsorb the template molecules so that a higher adsorption capacity could be achieved at a lower equilibrium concentration. As the equilibrium concentration increased, the adsorption of the MIPs continued to increase until a saturated adsorption capacity of 36.09 mg/g was reached. In contrast, the adsorption of template molecules by the NIPs was mainly nonspecific, with a low adsorption rate and a low adsorption capacity. As the equilibrium concentration increased, the adsorption capacity of the NIPs gradually increased, but the rate of increase was relatively slow; therefore, the adsorption isotherm of the NIPs showed a low adsorption capacity and a relatively flat curve shape, with an adsorption capacity of 25.34 mg/g.

The Scatchard equation is an equation used to describe the interaction between adsorbed materials and imprinted molecules in the adsorption process. The analysis of adsorption isotherm data with this equation is helpful in obtaining information about the affinity of adsorption sites and the maximum adsorption capacity [22,23]. The specific data are shown in Table 2 and Figure S2. Low K_D_ and high Q_max_ values are the key constants that determine the specificity of an imprinted material, indicating a high binding affinity and strong adsorption capacity. The MIPs and NIPs exhibited two different Scatchard plots. The data points of the MIPs were divided into two groups, with each corresponding to one kind of adsorption site. This suggested that there were two different types of adsorption sites in the MIPs with different affinities and adsorption capacities, which was probably due to the formation of multiple imprinted holes of different sizes and shapes in the MIPs during imprinting. A smaller dissociation constant indicated more stable adsorption, indicating a stronger interaction between the imprinted molecules and the MIPs. The Scatchard equation of the NIPs was linear, which indicated that the functional monomers of NIPs were distributed on the surface without significant site differences, and their adsorption behavior was that of a monolayer, which did not lead to the formation of special imprinted holes. In addition, it can be seen in the table that the maximum adsorption capacity of the MIPs was larger than that of the NIPs. Although the dissociation constant K_D_ of the high-affinity site was slightly smaller than that of the NIPs, the Q_max_ value of the MIPs was still larger. The reason for this may be related to the imprinted molecules. Some compounds may have directly bound to nonspecific binding sites on the surface without entering the imprinted holes, but the MIPs still had a larger value of Q_max_ due to the imprinted holes. 

### 2.3. Comparison of the MIPs’ Selectivity for the Adsorption of Flavonoids 

**Figure 7:**
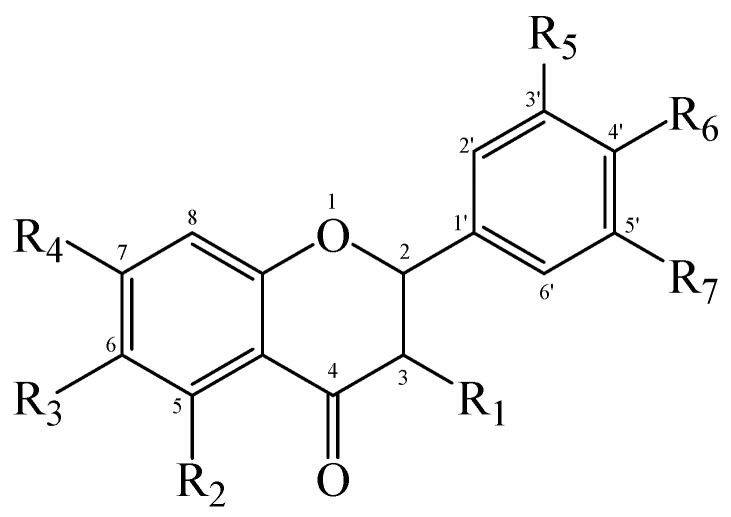
Structure of a typical flavonoid (R_1_~R_7_ = −H/−OH).

Six flavonoid compounds—chrysin, baicalin, fisetin, kaempferol, quercelin, and myricetin, which contain 2-phenylchromogenone but differ in their numbers of hydroxyl groups—were selected in this study. The adsorption properties of the MIPs on this flavonoid compound were studied. We explored the effects of the number and sites of hydrogen bonds on material selectivity by comparing the values of Q, IF, and other data on the MIPs for different flavonoids. The structures of the flavonoid compounds are shown in Figure 7, and Table 3 shows the adsorption performance data of the material for each compound. The adsorption capacity selection coefficient R_s_ indicates that the material tended to the binding ability of the compounds in the solvent. The larger the value of R_s_, the smaller the ability of the material to adsorb to the compounds.

It can be seen in the table that the material had the strongest adsorption ability for the template molecule myricetin, which was because myricetin provided more hydroxyl groups to interact with the material, which obviously improved the adsorption capacity. The R_s_ values of baicalein and quercetin reached 2.17 and 2.90, respectively, indicating that the binding abilities of these two compounds to the MIPs in the solvent were weak, which was mainly due to steric hindrance. Baicalein had three adjacent hydroxyl groups, which affected the adsorption efficiency, as well as quercetin. At the same time, it can be seen that myricetin had the highest absorbance but not the highest IF, which was primarily due to the hydroxyl site of the compound. For example, the benzene ring of myricetin had three adjacent hydroxyl groups (R_5_, R_6_, R_7_). In addition, different hydroxyl groups had different hydrogen bond strengths with functional monomers, such as R_2_, which were more likely to lose protons and form hydrogen bonds with functional monomers due to conjugation with neighboring carbonyl groups. At the same time, the O atom of meta R_4_ and R_6_ on the benzene ring was more electronegative. In addition, the ortho position of R_1_ had a benzene ring, which also made it easier to form hydrogen bonds. It can be seen that fisetin and kaempferol both had higher Q and larger IF values, while quercetin also had hydroxyl groups at the corresponding sites, but due to the steric effect of R_5_ and R_6_, the Q and IF values were lower. In addition, too many hydroxyl groups would also lead to an increase in the amount of nonspecific binding, which was one of the reasons for why myricetin was the template molecule with the largest number of effective hydroxyl groups that were capable of hydrogen bonding, but its IF was small. Finally, the three-dimensional configuration of a compound provides it with more action sites in different directions, which is more conducive to the improvement of the IF. For example, despite having no steric hindrance effect, chrysin had an R_2_ and R_4_ for two effective hydroxyl groups. The adsorption capacity was relatively high, but the IF value was the lowest, which was because chrysin only had action sites on one side. Therefore, the adsorption effect of the MIPs on flavonoid compounds was caused by many factors, among which the electronegativity of the oxygen atom on the hydroxyl group was one of the most important.

## 3. Materials and Methods

### 3.1. Reagents

Myricetin was purchased from Meryer (Shanghai, China). Chrysin was purchased from Macklin (Shanghai, China). Quercetin, fisetin, kaempferol, and baicalin were obtained from Aladdin Reagent (Shanghai, China). MAA and AIBN were obtained from Adamas (Shanghai China). ACN, MeOH, ethanol, glacial acetic acid, ammonia solution (25% *w*/*w*), and THF were purchased from Chron Chemicals (Chengdu, China). Tetraethyl orthosilicate (TEOS) and KH-570 were purchased from Sigma-Aldrich (St. Louis, MO, USA). Ultrapure water was purchased from Watsons (Hong Kong, China).

### 3.2. Apparatus and Instrument

A Shimadzu SPD-20A liquid chromatograph system (Shimadzu Corporation, Kyoto, Japan) and Shimadzu C18 column (250 × 4.6 mm, particle size, 5 μm) were used. The mobile phase consisted of methanol and water, and gradient elution was performed with methanol from 50% to 85%, 1.0 mL min^−1^, and a detection wavelength of 270 nm. All mobile phases were filtered through a 0.22 µm membrane filter before use. Standard samples were dissolved in HPLC-grade MeOH and injected in a volume of 10 µL. The characterization of MIPs and NIPs was performed using a Zeiss Sigma 500 scanning electron microscope (SEM, Spelle, Germany), FT-IR (Bruker, Billerica, MA, USA), and BET (Quantachrome, Boynton Beach, FL, USA).

### 3.3. Preparation of SiO_2_@C=C

The support material in this study was SiO_2_ grafted with double bonds, and the main synthesis steps were as follows: First, 114 mL of absolute ethanol and 11.4 mL of tetraethyl orthosilicate were mixed in a beaker, and then 50 mL of absolute ethanol, 76.5 mL of water, and 7.6 mL of ammonia were mixed in a beaker. Then, the two groups of solutions were quickly mixed and continuously stirred at a constant speed for 6 h. After centrifugation, the material was washed until neutral with absolute ethanol and dried in a vacuum-drying oven at 50 °C for 24 h to obtain SiO_2_ material. Following this, 1.0 g of SiO_2_ was added to 20 mL of pure water and 80 mL of absolute ethanol, and the mixture was sonicated and dispersed for 15 min. Under the condition of nitrogen protection, 3 mL of ammonia water and 4 mL of silane coupling agent KH-570 were slowly added, and the reaction was stirred at 65 °C for 24 h. After centrifugation, the material was washed to neutral with absolute ethanol, dried in a vacuum-drying oven at 50 °C for 24 h, and then sealed for use.

### 3.4. Preparation of Molecularly Imprinted Polymers 

The polymerization process of the MIPs was mainly as follows: Firstly, 0.04 mmol myricetin, 0.20 mmol MAA, and 20 mL porogen ACN were successively added to the beaker and placed in a 4 °C refrigerator for prepolymerization for 12 h. Then, SiO_2_@C=C (50 mg), EGDMA (1.6 mmol), and AIBN (30 mg) were added, purged under nitrogen, and sealed, and the reaction was carried out at 60 °C for 24 h. Polymerized material was obtained after centrifugation. Template molecules were eluted with methanol/acetic acid (9:1, *v*/*v*) until no template molecules were detected, and then the acid was washed with methanol. Finally, the samples were dried in a vacuum-drying oven at 50 °C for 24 h to obtain MIPs. NIPs were prepared using the same procedure but without the template molecules.

### 3.5. Properties of the Imprinted Material

The adsorption capacity is one of the main indicators used to measure the properties of adsorbents. By comparing the adsorption capacities of different adsorbents, their adsorption capacities can be evaluated, their adsorption mechanisms can be revealed, and the adsorption conditions can be optimized [24,25]. Adsorption capacity calculations are usually based on a similar principle to that used for conventional sorbents, that is, by measuring the change in concentration of the target molecule—usually the template molecule or its analogs—in solution before and after adsorption. The following formula was used to calculate the adsorption capacity:

(1) $$Q=\frac{C_{0}-C_{e}}{M}V,$$

where *Q* (mg/g) is the amount of adsorption per unit mass of adsorbent, *C*_0_ (mg/L) is the initial concentration of the solute in the solution before adsorption, and *C_e_* (mg/L) is the equilibrium concentration of the solute in the solution after adsorption. *V* (L) is the volume of the solution, and *M* (mg) is the mass of the adsorption material. All of the Q values in the experiment were calculated using Equation (1).

The Scatchard equation is an equation used to describe the interaction between adsorbents and adsorbents during adsorption. It is commonly used to analyze adsorption isotherm data to obtain information about the affinity of an adsorption site, the maximum adsorption capacity, and the homogeneity of the adsorption site. The following is the formula for the Scatchard equation:

(2) $$\frac{Q}{C}=\frac{Q_{max}-Q}{K_{D}}$$

where *Q* (mg/g) is the amount of adsorption at equilibrium (adsorbed mass per unit mass of adsorbent), *C* (mg/L) is the adsorbate concentration at equilibrium, *Q*_max_ (mg/g) is the maximum adsorption capacity (the maximum adsorption mass per unit mass of adsorbent), and K_D_ (mg/L) is the dissociation constant and represents the ease with which the adsorbate dissociates from the adsorbent.

### 3.6. Adsorption Experiments for MIPs and NIPs

In order to verify the adsorption properties of the materials, pH experiments, solvent adsorption experiments, adsorption kinetics experiments, and static adsorption experiments were carried out. The initial concentration of all adsorption solvents was 50 μg/mL, the concentration of adsorption materials was 2 mg/mL, and the adsorption conditions were shaking at 298 K and 120 rpm for two hours. All sample solutions were determined using HPLC and filtered through a 0.22 μm filter membrane prior to determination.

## 4. Conclusions

Through a series of adsorption experiments on MIPs prepared with myricetin as the template molecule, the difference in the adsorption capacity of a series of flavonoids was discussed, and the influence of MIPs on the adsorption effect and selectivity of flavonoids via hydrogen bonding was verified. The results showed that the MIPs made of the polyhydroxyl template had the strongest adsorption capacity for the template molecule, but they had weaker adsorption capacity for other derivatives with the same parent nucleus, such as baicalein and quercetin, and they had higher adsorption capacity and a larger IF for fisetin and kaempferol. The MIPs showed different selectivity to various flavonoids with the same parent nucleus. Although it is generally believed that this is determined by many factors, such as steric hindrance, compound structure, and material proportions, the electronegativity of the oxygen atom on the hydroxyl group of the compound is the most important, which led to the electronegativity of the specific hydroxyl group of 2-phenylchromogen playing a decisive role. Flavonoids with different hydroxyl groups in different numbers and directions have different hydrogen bond strengths with functional monomers. For example, the R_2_, R_4_ and R_1_ on 2-phenylchromogenone have stronger electronegativity, and the hydroxyl group is also more likely to form hydrogen bonds, and those bonds are more likely to be stronger. 

This material was able to efficiently separate individual target compounds from complex mixtures by identifying the flavonoids, which is of great value in practical applications, such as in pharmaceutical preparation, natural product extraction, and food safety testing. Most previous studies focused on the design and synthesis of MIPs for a specific compound, but there is a lack of discussion of the recognition mechanisms of classes of compounds [26,27]. We implemented a selective recognition strategy based on the strength, direction, and position of the hydrogen bond interactions, and this will be of great significance for the further study of molecular imprinting materials for compounds with the same parent core and different hydroxyl groups or other functional groups. Based on the results and the potential for development, the following suggestions can be made: (1) exploring different template molecules: More compounds can be explored rather than only the same kinds of compounds. (2) Studying new functional monomers: Functional monomers containing specific functional groups will improve the selectivity and adsorption performance of MIPs and expand their application range. (3) Carrying out computational analysis: Computational analysis will provide more information and help in the design and synthesis of MIPs. 

## Figures and Tables

**Table 1:** Optimization parameters for the preparation of surface-imprinted polymers.

	TM (mml)	FM (mmol)	Crosslinking Agent (mmol)	Pore-Forming Agent (mL)			IF
MIPs	Myricetin	MAA	EDGMA	MeOH	THF	ACN	Q_MIP_/Q_NIP_
MIP 1	0.04	0.04	1.20			30	1.03
MIP 2	0.04	0.20	1.20			30	1.85
MIP 3	0.04	0.40	1.20			30	1.43
MIP 4	0.04	0.60	1.20			30	1.08
MIP 5	0.04	0.20	0.40			30	0.49
MIP 6	0.04	0.20	0.80			30	1.02
MIP 7	0.04	0.20	1.60			30	1.37
MIP 8	0.04	0.20	1.20		30		1.26
MIP 9	0.04	0.20	1.20	30			1.14

TM, template; FM, functional monomer; MAA, methacrylic acid; EGDMA, ethylene dimethacrylate; MeOH, methanol; THF, tetrahydrofuran; ACN, acetonitrile; IF, imprinting factor.

**Table 2:** Dissociation constant (K_D_) and maximum adsorption capacity (Q_max_) as determined using Scatchard analyses.

	Low-Affinity Sites		High-Affinity Sites	
	K_D_ (mg/L)	Q_max_ (mg/g)	K_D_ (mg/L)	Q_max_ (mg/g)
MIP	990.10	268.49	140.84	59.08
NIP	122.70	42.39	/	/

**Table 3:** Flavonoids used in the adsorption studies.

Flavonoids	Chemical Structure	Hydroxyl Number	Hydroxyl Site	Q (mg/g)	R_s_^a^	Imprinting Factor
Chrysin	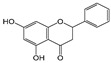	2	R_2_, R_4_	7.42	1.22	1.78
Baicalin	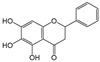	3	R_2_, R_3_, R_4_	4.17	2.17	2.50
Fisetin	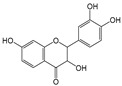	4	R_1_, R_4_, R_5_, R_6_	8.78	1.03	3.37
Kaempferol	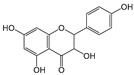	4	R_1_, R_2_, R_4_, R_6_	6.79	1.33	2.65
Quercelin	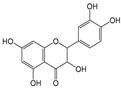	5	R_1_, R_2_, R_4_, R_5_, R_6_	3.12	2.90	2.00
Myricetin	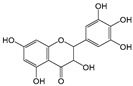	6	R_1_, R_2_, R_4_ R_5_, R_6_, R_7_	9.04	1	1.86

^a^ R_s_ = Q_TM_/Q; Q_TM_, the amount of template molecules adsorbed; Q, the amount of the corresponding compound adsorbed.

## Data Availability

The data that support the findings of this study are available from the corresponding author upon reasonable request.

## Supplementary Materials

The following supporting information can be downloaded at: https://www.mdpi.com/article/10.3390/molecules29061292/s1, Figure S1: Adsorption of the MIPs at a range of pH to flavonoids; Figure S2: Scatchard analysis curves of the MIP and NIP; Figure S3: Scanning electron microscope images of the MIP and NIP.
