# Supplementary material for: Study on the Selectivity of Molecular Imprinting Materials Determined through Hydrogen Bonding on Template Molecular Structures of Flavonoids
Source: Molecules. 2024 Mar 14;29(6):1292. doi: 10.3390/molecules29061292 (PMC10975109; doi:10.3390/molecules29061292)
Supplement: Supplementary file 1 [file molecules-29-01292-s001.zip › molecules-2884887-supplementary.pdf]

Supplementary Figures

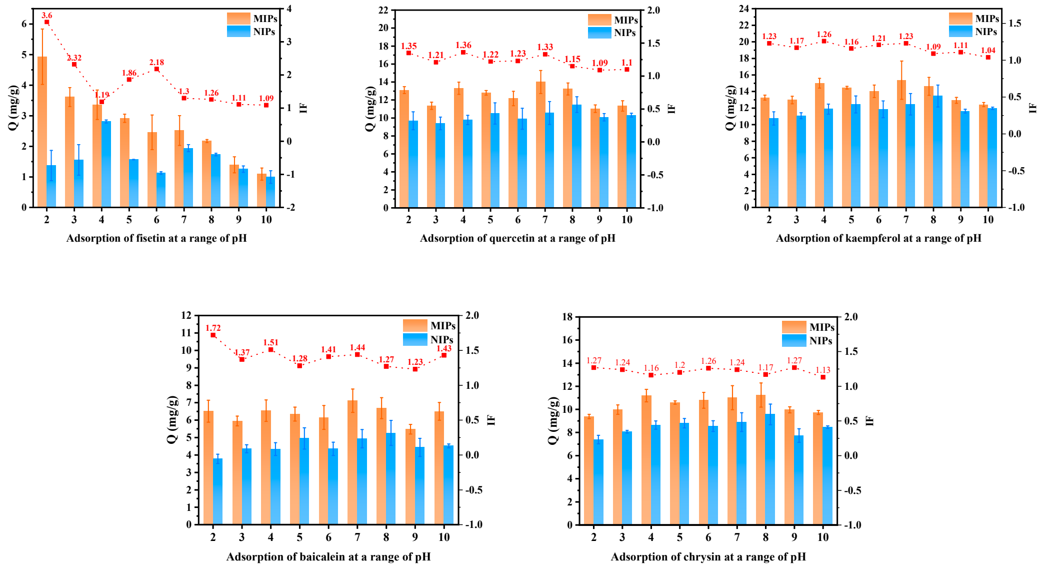

Supplementary Figure S1. Adsorption of the MIPs at a range of pH to flavonoids

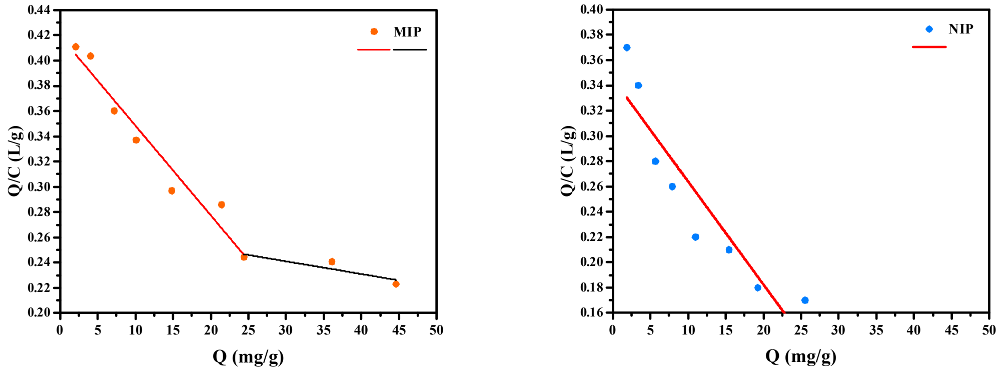

Supplementary Figure S2. Scatchard analysis curves of the MIP and NIP

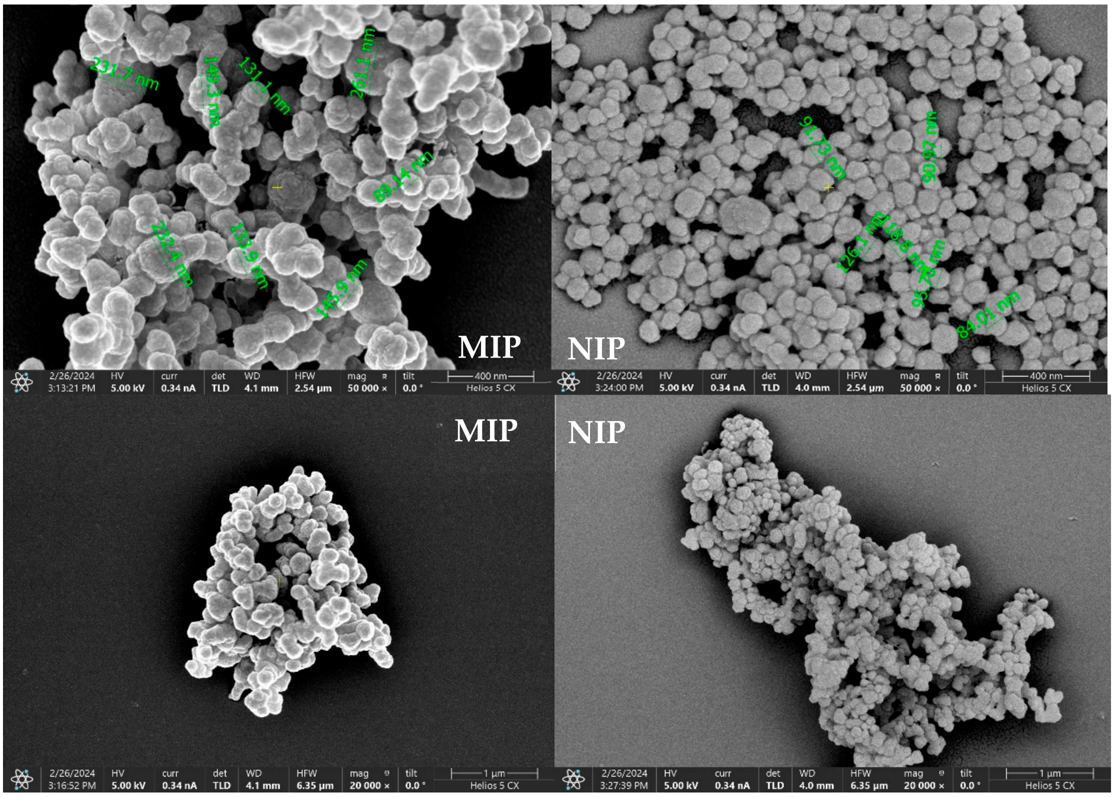

Supplementary Figure S3. Scanning electron microscope images of the MIP and NIP.
